# Three-dimensional localization spectroscopy of individual nuclear spins with sub-Angstrom resolution

**DOI:** 10.1038/s41467-018-07121-0

**Published:** 2018-11-08

**Authors:** J. Zopes, K. S. Cujia, K. Sasaki, J. M. Boss, K. M. Itoh, C. L. Degen

**Affiliations:** 1 Department of Physics, ETH Zurich, Otto Stern Weg 1, 8093 Zurich, Switzerland; 2 School of Fundamental Science and Technology, Keio University, Yokohama, 223-8522 Japan

## Abstract

Nuclear magnetic resonance (NMR) spectroscopy is a powerful method for analyzing the chemical composition and molecular structure of materials. At the nanometer scale, NMR has the prospect of mapping the atomic-scale structure of individual molecules, provided a method that can sensitively detect single nuclei and measure inter-atomic distances. Here, we report on precise localization spectroscopy experiments of individual ^13^C nuclear spins near the central electronic sensor spin of a nitrogen-vacancy (NV) center in a diamond chip. By detecting the nuclear free precession signals in rapidly switchable external magnetic fields, we retrieve the three-dimensional spatial coordinates of the nuclear spins with sub-Angstrom resolution and for distances beyond 10 Å. We further show that the Fermi contact contribution can be constrained by measuring the nuclear *g*-factor enhancement. The presented method will be useful for mapping atomic positions in single molecules, an ambitious yet important goal of nanoscale nuclear magnetic resonance spectroscopy.

## Abstract (web summary)

Electron spins in nitrogen vacancy centres are perturbed by nearby nuclear spins, making it possible to infer some of the nuclear spins' properties. Here the authors demonstrate a technique that can determine the location of nuclear spins in three-dimensional space using only one electron spin.

## Introduction

One of the visionary goals of nanoscale quantum metrology with NV centers is the structural imaging of individual molecules, for example proteins, that are attached to the surface of a diamond chip^1^. By adapting and extending measurement techniques from nuclear magnetic resonance (NMR) spectroscopy, the long-term perspective is to reconstruct the chemical species and three-dimensional location of the constituent atoms with sub-Angstrom resolution^2,3^. In contrast to established structural imaging techniques like X-ray crystallography, cryo-electron tomography, or conventional NMR, which average over large numbers of target molecules, only a single copy of a molecule is required. Conformational differences between individual molecules could thus be directly obtained, possibly bringing new insights about their structure and function.

In recent years, first experiments that address the spatial mapping of nuclear and electron spins with NV-based quantum sensors have been devised. One possibility is to map the position into a spectrum, as it is done in magnetic resonance imaging. For nanometer-scale imaging, this requires introducing a nanomagnet^4–6^. Another approach is to exploit the magnetic gradient of the NV center’s electron spin itself, whose dipole field shifts the resonances of nearby nuclear spins as a function of distance and internuclear angle. Refinements in quantum spectroscopic techniques have allowed the detection of up to 8 individual nuclear spins^7,8^ as well as of spin pairs^9–11^ for distances of up to ~30 Å^12,13^. Due to the azimuthal symmetry of the dipolar interaction, however, these measurements can only reveal the radial distance *r* and polar angle *θ* of the inter-spin vector **r** = (*r*, *θ*, *ϕ*), but are unable to provide the azimuth *ϕ* required for reconstructing three-dimensional nuclear coordinates. One possibility for retrieving *ϕ* is to change the direction of the static external field^12^, however, this method leads to a mixing of the NV center’s spin levels which suppresses the ODMR signal^14^ and shortens the coherence time^15^. Other proposed methods include position-dependent polarization transfer^16^ or combinations of microwave and radio-frequency fields^17–19^.

Here we demonstrate three-dimensional localization of individual, distant nuclear spins with sub-Angstrom resolution. To retrieve the missing angle *ϕ*, we combine a dynamic tilt of the quantization axes using a high-bandwidth microcoil with high resolution correlation spectroscopy^20,21^. Our method provides the advantage that manipulation and optical readout of the electronic spin can be carried out in an aligned external bias field. This ensures best performance of the optical readout and the highest magnetic field sensitivity and spectral resolution of the sensor.

## Results

### Theory

We consider a nuclear spin *I* = 1/2 located in the vicinity of a central electronic spin *S* = 1 with two isolated spin projections *m*_*S*_ = {0, −1}. The nuclear spin experiences two types of magnetic field, a homogeneous external bias field *B*_0_ (aligned with the quantization axis **e**_*z*_ of the electronic spin), and the local dipole field of the electronic spin. Because, the electronic spin precesses at a much higher frequency than the nuclear spin, the latter only feels the static component of the electronic field, and we can use the secular approximation to obtain the nuclear free precession frequencies,

1 $$f_{m_S} = \frac{1}{{2\pi }}\left\| { - \gamma _{\mathrm{n}}{\bf{B}}_{{\mathrm{tot}}}} \right\| = \frac{1}{{2\pi }}\left\| { - \gamma _{\mathrm{n}}B_0{\bf{e}}_z + m_S{\bf{A}}_z({\bf{r}})} \right\|.$$

**Fig. 1:**
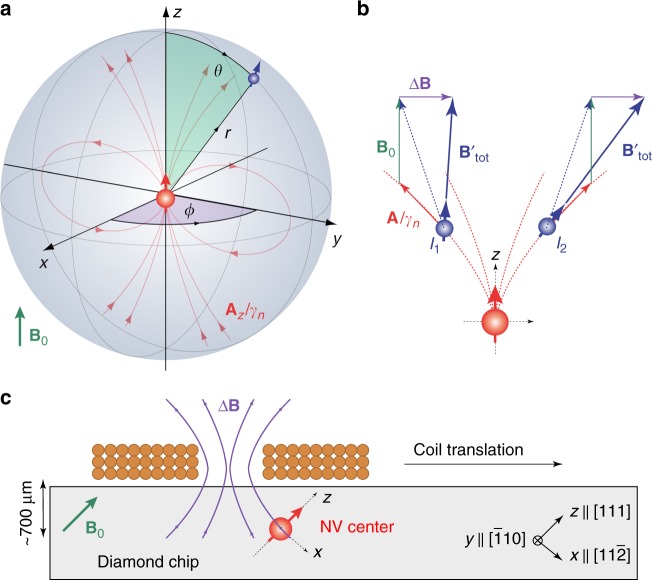
Coordinate systems for spins and magnetic fields. **a** Reference frame of the central nitrogen-vacancy (NV) sensor spin (red) with a nuclear spin (blue) located at the three-dimensional position **r** = (*r*, *θ*, *ϕ*). The quantization axis of the NV center defines the *z*-axis. The hyperfine field of the NV spin (red field lines) provides the magnetic field gradient for imaging. **b** Sketch of two nuclear spins *I*_1_ and *I*_2_ experiencing the same hyperfine interaction (red) [Eq. (2)]. Application of a transverse field Δ**B** (purple) reduces (*I*_1_) or increases (*I*_2_) the total magnetic field ${\bf{B}}_{{\mathrm{tot}}}^\prime$ (blue) experienced by the nuclear spins depending on the *ϕ* angle, allowing us to discriminate the nuclear locations. *B*_0_ is the static external field (green). **c** Geometry of the experimental setup in the laboratory frame of reference. A small solenoid on top of the diamond chip provides a rapidly switchable magnetic field Δ**B**. To change the vector orientation of Δ**B**, we translate the coil over the diamond

Here, *γ*_n_ is the nuclear gyromagnetic ratio and

2 $$\begin{array}{*{20}{l}} {{\bf{A}}_z({\bf{r}})} \hfill & = \hfill & {\underline {\underline {\bf{A}} } ({\bf{r}}) \cdot {\bf{e}}_z = \left( {A_{xz},A_{yz},A_{zz}} \right)} \hfill \\ {} \hfill & = \hfill & {\left( {a_ \bot {\kern 1pt} {\mathrm{cos}}(\phi ),a_ \bot {\kern 1pt} {\mathrm{sin}}(\phi ),a_{||}} \right)} \hfill \end{array}$$

is the secular hyperfine vector of the hyperfine tensor $\underline {\underline {\bf{A}} } ({\bf{r}})$ that gives rise to the hyperfine magnetic field *m*_*S*_**A**_*z*_(**r**)/*γ*_n_ (see Fig. 1b).

To obtain information about the distance vector **r**, a standard approach is to measure the parallel and transverse components of the hyperfine vector, *a*_||_ = *A*_*zz*_ and $a_ \bot = \left( {A_{xz}^2 + A_{yz}^2} \right)^{1/2}$, and to relate them to the field of a point dipole,

3 $$a_{||} = \frac{{\mu _0\gamma _{\mathrm{e}}\gamma _{\mathrm{n}}\hbar }}{{4\pi r^3}}\left( {3{\kern 1pt} {\mathrm{cos}}^2\theta - 1} \right) + a_{{\mathrm{iso}}},$$

4 $$a_ \bot = \frac{{\mu _0\gamma _{\mathrm{e}}\gamma _{\mathrm{n}}\hbar }}{{4\pi r^3}}3{\kern 1pt} {\mathrm{sin}}{\kern 1pt} \theta {\kern 1pt} {\mathrm{cos}}{\kern 1pt} \theta ,$$

where *μ*_0_ = 4*π* × 10^−7^ TmA^−1^ is the vacuum permeability, *ħ* = 1.054 × 10^−34^ Js is the reduced Planck constant, |*γ*_e_| = 2*π* × 28 GHz T^−1^ is the electron gyromagnetic ratio, and where we have included a Fermi contact term *a*_iso_ (set to zero for now) for later discussion. Experimentally, the parallel projection *a*_||_ can be inferred from the precession frequencies $f_{m_S}$ using Eq. (1), and the transverse projection *a*_⊥_ can be determined by driving a nuclear Rabi rotation via the hyperfine field of the central spin and measuring the rotation frequency^21^. Once *a*_||_ and *a*_⊥_ are known, Eqs. (3) and (4) can be used to extract the distance *r* and polar angle *θ* of the distance vector **r** = (*r*, *θ*, *ϕ*). Due to the rotational symmetry of the hyperfine interaction, however, knowledge of *a*_||_ and *a*_⊥_ is insufficient for determining the azimuth *ϕ*.

To break the rotational symmetry and recover *ϕ*, we apply a small transverse magnetic field Δ**B** during the free precession of the nuclear spin. Application of a transverse field tilts the quantization axes of the nuclear and electronic spins. The tilting modifies the hyperfine coupling parameters *a*_||_ and *a*_⊥_ depending on the angle between Δ**B** and **A**_*z*_, which in turn shifts the nuclear precession frequencies $f_{m_S}$. To second-order in perturbation theory, the *m*_*S*_-dependent precession frequencies are given by:^22^

5 $$\begin{array}{*{20}{l}} {f_{m_S}} \hfill & = \hfill & {\frac{1}{{2\pi }}\left\| { - \gamma _{\mathrm{n}}{\bf{B}}_{{\mathrm{tot}}}^\prime } \right\|} \hfill \\ {} \hfill & = \hfill & {\frac{1}{{2\pi }}\left\| { - \gamma _{\mathrm{n}}B_0{\bf{e}}_z - \gamma _{\mathrm{n}}\left( {1 + \alpha (m_S)} \right){\mathrm{\Delta }}{\bf{B}} + m_S{\bf{A}}_z({\bf{r}})} \right\|,} \hfill \end{array}$$

where *α*(*m*_*S*_) is a small enhancement of the nuclear *g*-factor. The enhancement results from non-secular terms in the Hamiltonian that arise due to the tilting of the electronic quantization axis, and is given by^22^

6 $$\alpha \left( {m_S} \right) \approx \left( {3\left| {m_S} \right| - 2} \right)\frac{{\gamma _{\mathrm{e}}}}{{\gamma _{\mathrm{n}}D}}\left( {\begin{array}{*{20}{c}} {A_{xx}} & {A_{xy}} & {A_{xz}} \\ {A_{yx}} & {A_{yy}} & {A_{yz}} \\ 0 & 0 & 0 \end{array}} \right).$$

Here *D* = 2*π* × 2.87 GHz is the ground-state zero-field splitting of the NV center. By measuring the shifted frequencies $f_{m_S}$ and comparing them to the theoretical model of Eqs. (5) and (6), we can then determine the relative *ϕ* angle between the hyperfine vector and Δ**B**.

### Experimental setup

We experimentally demonstrate three-dimensional localization spectroscopy of four ^13^C_1–4_ nuclei adjacent to three distinct NV centers. NV_1_ is coupled to two ^13^C spins, while NV_2_ and NV_3_ are each coupled to a single ^13^C spin. For readout and control of the NV center spin, we use a custom-built confocal microscope that includes a coplanar waveguide and a cylindrical permanent magnet for providing an external bias field of *B*_0_ ~ 10 mT applied along the NV center axis **e**_*z*_. Precise alignment of the bias field is crucial for our experiments and is better than 0.3° (Methods section).

To dynamically tilt the external field we implement a multi-turn solenoid above the diamond surface (Fig. 1d). The coil produces ~2.5 mT field for 600 mA of applied current and has a rise time of ~2 μs. We calibrate the vector magnetic field of the coil with an absolute uncertainty of less than 15 μT in all three spatial components using two other nearby NV centers with different crystallographic orientations (ref. ^23^ and Methods section).

### Mapping of *r* and *θ*

**Fig. 2:**
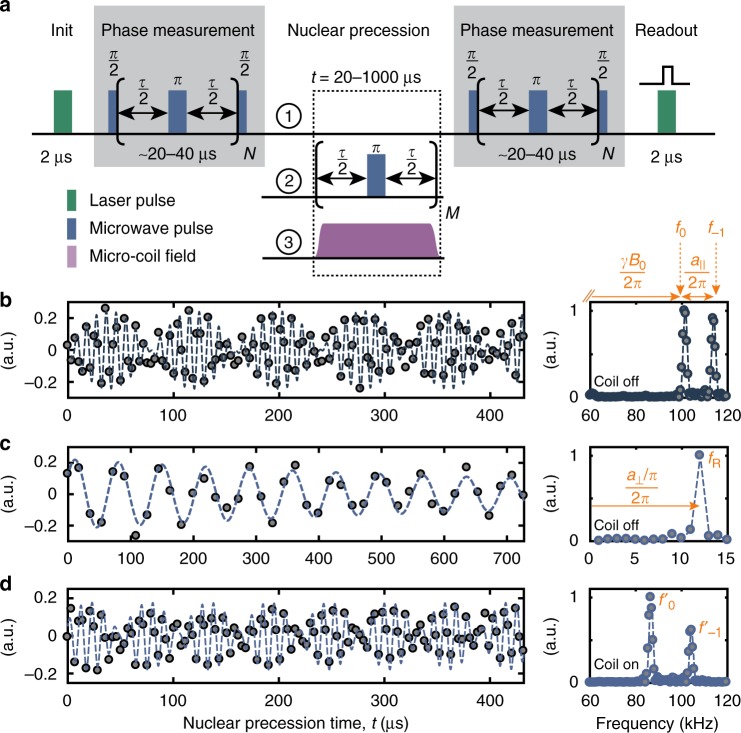
Implementation of three-dimensional localization spectroscopy. **a** Correlation spectroscopy protocol. By correlating two phase measurements we trace out the precession of the target nuclear spin(s) under different NMR sequences. Phase measurements are implemented by a Carr–Purcell–Meiboom–Gill (CPMG) train of microwave *π* pulses applied to the central electronic spin, where *τ* ≈ [2(*f*_0_ + *f*_−1_)]^−1^. Laser pulses are used to polarize and readout the electronic spin. Repetitions are *N* = 4–8 and *M* = *t*/*τ*. Experimental parameters for all measurements are collected in Supplementary Tables 2–9. **b** Free precession signal of the nuclear spin as a function of time *t*, using sequence 1. Right panel shows the corresponding power spectrum. The two frequencies *f*_0_ and *f*_−1_ are approximately equal to *γ*_n_*B*_0_/(2*π*) and (*γ*_n_*B*_0_ + *a*_||_)/(2*π*), respectively, see text. **c** Application of periodic *π* pulses on the NV center during *t* (sequence 2) causes a Rabi nutation of the nuclear spin, whose oscillation frequency *f*_R_ is approximately equal to (*a*_⊥_/*π*)/(2*π*). **d** Activation of a transverse microcoil field Δ**B** during the nuclear precession (sequence 3) leads to shifted frequencies $f_0^\prime$ and $f_{ - 1}^\prime$. All measurements were conducted on ^13^C_1_. Extracted frequencies are listed in Table 1

We begin our 3D mapping procedure by measuring the parallel and perpendicular hyperfine coupling constants using conventional correlation spectroscopy^21^ with no coil field applied, Δ**B** = 0 (Fig. 2). The parallel coupling *a*_||_ is determined from a free precession experiment (sequence 1 in Fig. 2) yielding the frequencies *f*_0_ and *f*_−1_ (Fig. 2b). The coupling constant is then approximately given by *a*_||_/(2*π*) ≈ *f*_−1_ − *f*_0_. The transverse coupling *a*_⊥_ is obtained by driving a nuclear Rabi oscillation via the NV spin, using sequence 2, and recording the oscillation frequency *f*_R_, where *a*_⊥_/(2*π*) ≈ *πf*_R_ (Fig. 2c). Because, the Zeeman and hyperfine couplings are of similar magnitude, these relations are not exact and proper transformation must be applied to retrieve the exact coupling constants *a*_||_ and *a*_⊥_ (ref. ^21^ and Methods section). Once the hyperfine parameters are known, we can calculate the radial distance *r* = 8.58(1) Å and the polar angle *θ* = 52.8(1)° of the nuclear spin by inverting the point-dipole formulas (Eqs. 3, 4). The measurement uncertainties in *r* and *θ* are very small because correlation spectroscopy provides high precision estimates of both *a*_||_ and *a*_⊥_.

### Mapping of *ϕ*

In a second step, we repeat the free precession measurement with the coil field turned on (sequence 3), yielding a new pair of frequency values $f_0^\prime$, $f_{ - 1}^\prime$ (Fig. 2d). We then retrieve *ϕ* by computing theoretical values for $f_0^{{\mathrm{(th)}}}$, $f_{ - 1}^{{\mathrm{(th)}}}$ based on Eq. (5) and the calibrated fields in Table 1, and minimizing the cost function

7 $$\xi (\phi ) = \left[ {f_{ - 1}^\prime - f_0^\prime } \right] - \left[ {f_{ - 1}^{{\mathrm{(th)}}}(\phi ) - f_0^{{\mathrm{(th)}}}(\phi )} \right]$$

with respect to *ϕ*. To cancel residual shifts in the static magnetic field and improve the precision of the estimates, we compare the frequency difference between *m*_*S*_ states rather than the absolute precession frequencies.

**Table 1:** Data base of measured precession frequencies and calibrated external magnetic fields used to determine the 3D position of ^13^C_1_

Quantity	Value	Reference
*f*_0_, *f*_−1_	101.7(1), 114.2(1) kHz	Fig. 2b
*f*_R_	14.4(1) kHz	Fig. 2c
$f_0^\prime$, $f_{ - 1}^\prime$	88.3(3), 103.2(2) kHz	Fig. 2d
**B**_0_	(0.028, −0.056, 9.502) mT	Supplementary Tables 2 and 3
Δ**B**	(−1.715, 0.614, −1.547) mT	Supplementary Table 2 and 3

Five further measurements of $\left( {f_0^\prime ,f_{ - 1}^\prime } \right)$ were made to improve the localization accuracy (data given in Supplementary Tables 2, 3). Vector magnetic fields refer to the NV coordinate system defined in Fig. 1

**Fig. 3:**
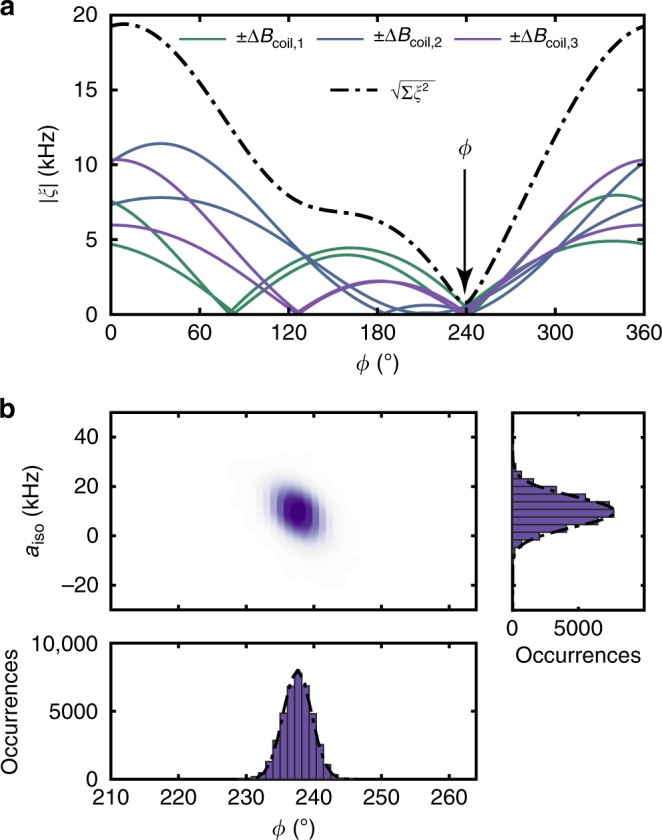
Determination of azimuth angle *ϕ* and Fermi contact contribution *a*_iso_ for ^13^C_1_. **a** Cost function |*ξ*(*ϕ*)| between observed and predicted precession frequencies, as defined in Eq. (7). Here *a*_iso_ = 0. Six measurements are shown for three spatial coil positions (solid curves) and opposite polarities of the coil current. The estimate for *ϕ* is given by the minimum of the squared cost functions ${\sum} \left| {\xi (\phi )} \right|^2$ of the six measurements (dash-dotted curve). **b** Scatter plot of maximum likelihood estimates of *ϕ* and *a*_iso_ obtained by Monte Carlo error propagation. The plot is generated from 4 × 10^4^ scatter points, where each point is the result of minimizing ${\sum} \left| {\xi \left( {\phi ,a_{{\mathrm{iso}}}} \right)} \right|^2$ for a different Monte Carlo sampling. Histograms for *ϕ* (bottom) and *a*_iso_ (right) are obtained by integrating the 2D scatter plot along the vertical or horizontal direction, respectively. Corresponding plots for ^13^C_2–4_ are given in Supplementary Figs. 3–5

In Fig. 3a, we plot |*ξ*(*ϕ*)| for three different coil positions and opposite coil currents for ^13^C_1_. We use several coil positions because a single measurement has two symmetric solutions for *ϕ*, and also because several measurements improve the overall accuracy of the method. The best estimate *ϕ* = 239(2)° is then given by the least squares minimum of the cost functions (dash-dotted line in Fig. 3a). To obtain a confidence interval for *ϕ*, we calculate a statistical uncertainty for each measurement by Monte Carlo error propagation taking the calibration uncertainties in **B**_0_ and Δ**B**, as well as the measurement uncertainties in the observed precession frequencies into account (Methods section). Values for all investigated ^13^C nuclei are collected in Supplementary Tables 2–9.

### Fermi contact interaction

Thus far we have assumed that the central electronic spin generates the field of a perfect point dipole. Previous experimental work^22,24^ and density functional theory (DFT) simulations^25,26^, however, suggest that the electronic wave function extends several Angstrom into the diamond host lattice. The finite extent of the spin density leads to two deviations from the point dipole model: modified hyperfine coupling constants *A*_*ij*_, and a non-zero Fermi contact term *a*_iso_. In the remainder of this study we estimate the systematic uncertainty to the localization of the nuclear spins due to deviations from the point dipole model.

We first consider the influence of the Fermi contact interaction, which arises from a non-vanishing NV spin density at the location of the nuclear spin. The Fermi contact interaction adds an isotropic term to the hyperfine coupling tensor, **A** + *a*_iso_**1**, which modifies the diagonal elements *A*_*xx*_, *A*_*yy*_, and *A*_*zz*_. DFT simulations^25,26^ indicate that *a*_iso_ can exceed 100 kHz even for nuclear spins beyond 7 Å. It is therefore important to experimentally constrain the size of *a*_iso_.

To determine *a*_iso_, one might consider measuring the contact contribution to the parallel hyperfine parameter *a*_||_, which is equal to *A*_*zz*_. This approach, however, fails because a measurement of *a*_||_ cannot distinguish between dipolar and contact contributions. Instead, we here exploit the fact that the gyromagnetic ratio enhancement *α* depends on *A*_*xx*_ and *A*_*yy*_, and hence *a*_iso_. To quantify the Fermi contact coupling we include *a*_iso_ as an additional free parameter in the cost function (7). By minimizing *ξ*(*ϕ*, *a*_iso_) as a joint function of *ϕ* and *a*_iso_ and generating a scatter density using Monte Carlo error propagation, we obtain maximum likelihood estimates and confidence intervals for both parameters (Fig. 3b). The resulting contact coupling and azimuth for nuclear spin ^13^C_1_ are *a*_iso_/(2*π*) = 9(8) kHz and *ϕ* = 238(2)°, respectively; data for ^13^C_2–4_ are collected in Table 2. Because the gyromagnetic ratio enhancement *α* is only a second-order effect, our estimate is poor, but it still allows us constraining the size of *a*_iso_. By subtracting the Fermi contact contribution from *a*_||_, we further obtain refined values for the radial distance and polar angle, *r* = 8.3(2) Å and *θ* = 58(4)°. Note that introducing *a*_iso_ as a free parameter increases the uncertainties in the refined *r* and *θ*, because the error in *a*_iso_ is large. This leads to disproportionate errors for distant nuclei where *a*_iso_ is small. Once nuclei are beyond a certain threshold distance, which we set to *r* = 10 Å in Table 2, it therefore becomes more accurate to constrain *a*_iso_ = 0 and apply the simple point dipole model.

**Table 2:** Measured hyperfine couplings and inferred 3D locations of ^13^C nuclei measured on three NV centers

	Experimental values						DFT values^26^						
Atom	*a*_||_/2*π* (kHz)	*a*_⊥_/2*π* (kHz)	*a*_iso_/2*π* (kHz)	*r* (Å)	*θ* (°)	*ϕ* (°)	Site	*a*_||_/2*π* (kHz)	*a*_⊥_/2*π* (kHz)	*a*_iso_/2*π* (kHz)	*r*_DFT_ (Å)	*θ*_DFT_ (°)	*ϕ*_DFT_ (°)
^13^C_1_	3.1(1)	44.5(1)	9(8)	8.3(2)	58(4)	238(2)	447	1.1	43.1	3.9	8.6	60^a^	240
^13^C_2_	119.0(1)	65.9(1)	19(15)	6.8(3)	19(3)	20(5)	25	98.4	65.8	−6.0	6.3	24^a^	30
^13^C_3_	18.5(1)	41.4(2)	3(4)	8.9(1)	43(4)	197(4)	47	30.1	45.8	1.5	8.7	37^a^	195
^13^C_4_	1.9(1)	19.2(1)	0^b^	11.47(1)	51.8(2)	34(4)	—^c^	—	—	—	—	—	—

Errors are one standard deviation and represent the confidence interval from the Monte Carlo error propagation according to Fig. 3b. DFT values are for the lattice site(s) whose calculated hyperfine couplings best match the experimental data

^a^Due to the inversion symmetry of the hyperfine interaction, our method cannot distinguish between sites in the upper and lower hemisphere; the table therefore lists min(*θ*_DFT_, 180° − *θ*_DFT_)

^b^Constrained to *a*_iso_ = 0

^c^No DFT data available

### Extended electronic wave function

**Fig. 4:**
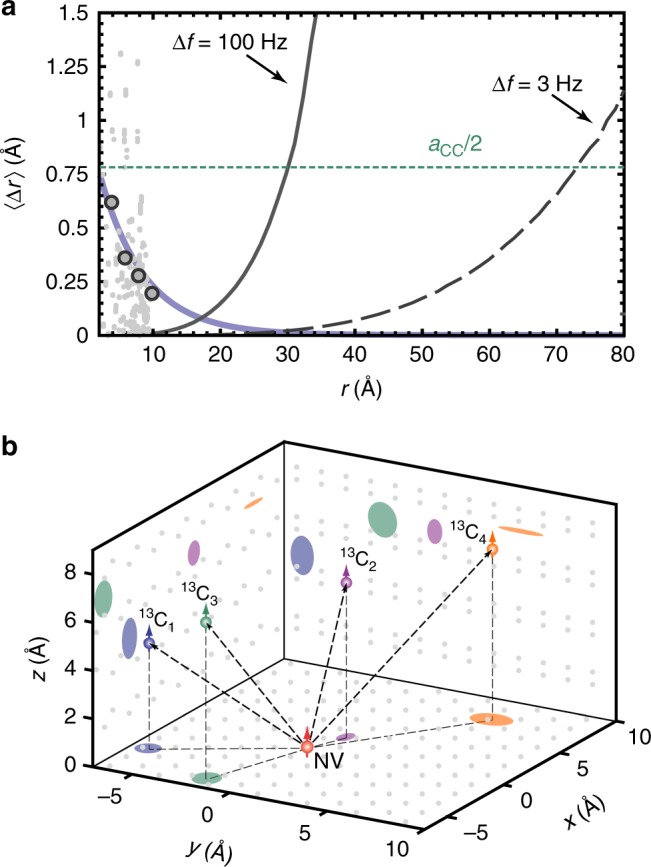
Three-dimensional localization of four ^13^C nuclear spins. **a** Average localization uncertainty 〈Δ*r*〉 as a function of radial distance *r* to the central spin. Gray dots represent the systematic error of the point-dipole approximation (see text), extracted for all lattice sites reported in the DFT calculation of ref. ^26^. Blue curve is an exponential fit to the median values (gray circles) of the gray dots in intervals of 2 Å. Black curves show the uncertainty of the frequency measurement, assuming a precision of 100 Hz (solid line, this study) and of 3 Hz^11, 38, 39^ (dashed line). Dotted horizontal line is one-half the diamond C–C bond length of *a*_C−C_ = 1.54 Å. **b** Reconstructed locations of the four distant nuclear spins ^13^C_1–4_. Shaded regions mark the 2*σ*-confidence area of the localization projected onto (*xy*, *yz*, *xz*)-planes of the coordinate system. Gray points represent carbon lattice positions projected onto the same planes. The origin is set to the expected center of gravity of the spin density at 2.29 Å from the nitrogen nucleus on the NV symmetry axis^25, 26^. Due to the inversion symmetry of the hyperfine interaction, our method cannot distinguish between sites in the upper and lower hemisphere; all ^13^C are therefore plotted in the upper hemisphere

The second systematic error in the position estimate results from the finite size of the NV center’s electronic wave function. Once the extent of the wave function becomes comparable to **r**, the anisotropic hyperfine coupling constants *A*_*ij*_ are no longer described by a point dipole, but require integrating a geometric factor over the sensor spin density^25^. While we cannot capture this effect experimentally, we can estimate the localization uncertainty from DFT simulations of the NV electron spin density. Following ref. ^26^, we convert the calculated DFT hyperfine parameters of 510 individual lattice sites to (*r*, *θ*) positions using the point-dipole formula (Eqs. 3, 4), and compute the difference to the DFT input parameters (*r*_DFT_, *θ*_DFT_). The result is plotted in Fig. 4a. We find that the difference 〈Δ*r*〉 = *r* − *r*_DFT_ decreases roughly exponentially with distance, and falls below 0.2 Å when *r* > 10 Å (gray dots and curve).

## Discussion

Figure 4b summarizes our study by plotting the reconstructed locations for all four carbon atoms in a combined 3D chart. The shaded regions represent the confidence areas of the localization, according to Table 2, projected onto the Cartesian coordinate planes. We note that the DFT simulations are in good agreement with our experimental results.

The accuracy of our present experiments is limited by deviations from the point-dipole model, which dominate for small *r* (Fig. 4a). For larger *r* ≳ 1 nm, this systematic uncertainty becomes negligible, and the localization imprecision is eventually dictated by the NMR frequency measurement. In the present study, which probed isolated ^13^C nuclei with a narrow intrinsic line-width, the frequency precision was limited by the accuracy of our detection protocol to ~100 Hz. This corresponds to a radial localization error of ~0.75 Å at a distance of *r* = 3 nm (solid black line in Fig. 4a and Supplementary Fig. 6). Improving the frequency precision to 3 Hz^11,27^ would extend this distance to *r* ~ 7 nm (dashed black line).

Our work demonstrates a basic strategy for mapping spatial positions of single nuclei in 3D with high precision. Extending these experiments to single molecules outside a diamond chip poses a number of additional challenges, and overcoming them will require the combination of several strategies. To isolate single molecules, they can be embedded in a spin-free matrix layer deposited on the diamond surface^28^ or immobilized by a linker chemistry^29^. Nuclear dipole interactions can be suppressed using homo- and heteronuclear decoupling^13^, taking advantage of the existing microcoil. Line-widths and spectral complexity can be further reduced by polarizing the constituent nuclei, and by spin dilution and isotope labeling of molecules^30^. Alternatively, measurements of inter-spin couplings will allow constraining the structure and size of molecules^31^. To sensitively detect very weakly coupled nuclei in distant molecules, spin precession can be recorded by repetitive weak measurements^32,33^. Further improvement of sensitivity is possible by optimizing the optical detection efficiency compared to our present setup^34^, and possibly by cryogenic operation^11^. How well these strategies will work we do not know at present, but we believe that the prospect of a general single-molecule MRI technique, which will have many applications in structural biology and chemical analytics, provides sufficient motivation to warrant these efforts.

## Methods

### Diamond sample

Experiments were performed on a bulk, electronic-grade diamond crystal from ElementSix with dimensions 2 × 2 × 0.5 mm with $\left\langle {110} \right\rangle$ edges and a $\left\langle {100} \right\rangle$ front facet. The diamond was overgrown with a layer structure of 20 nm enriched ^12^C (99.99%), 1 nm enriched ^13^C (estimated in-grown concentration ~5–10%) and a 5 nm cap layer of again enriched ^12^C (99.99%). Nitrogen-vacancy (NV) centers were generated by ion-implantation of ^15^N with an energy of 5 keV, corresponding to a depth of ~5–10 nm. After annealing the sample for NV formation, we had to slightly etch the surface (at 580 °C in pure O_2_) to remove persistent surface fluorescence. The intrinsic nuclear spin of the three NV centers studied in our experiments were confirmed to be of the ^15^N isotope. Further characterizations and details on the sample can be found in a recent study (sample B in ref. ^35^).

### Coordinate systems

In Supplementary Fig. 2a both laboratory and NV coordinate system are shown in a combined schematic. The laboratory coordinate system (*x*_Lab_, *y*_Lab_, *z*_Lab_) is defined by the normal vectors to the diamond faces, which lie along $\left\langle {110} \right\rangle$, $\left\langle {\bar 110} \right\rangle$ and $\left\langle {001} \right\rangle$, respectively. The reference coordinate system of the NV center is defined by its quantization direction, which is labeled *z*_NV_ and lies along $\left\langle {111} \right\rangle$. The *x*_NV_- and *y*_NV_-axis are pointing along the $\left\langle {11\bar 2} \right\rangle$ and $\left\langle {\bar 110} \right\rangle$ direction, respectively.

### Experimental apparatus

A schematic of the central part of the experimental apparatus is shown in Supplementary Fig. 1. The diamond sample is glued to a 200 μm thick glass piece and thereby held above a quartz slide with incorporated microwave transmission line for electron spin control. Below the quartz slide we placed a high numerical aperture (NA = 0.95) microscope objective for NV excitation with a 532 nm laser and detection using a single photon counting module (SPCM). We applied static, external magnetic bias fields with a cylindrical NdFeB permanent magnet (not shown in Supplementary Fig. 1). The magnet is attached to a motorized, three-axis translation stage. The NV control pulses were generated by an arbitrary waveform generator (Tektronix, AWG5002C) and upconverted by I/Q mixing with a local oscillator to the desired ~2.6 GHz.

### Planar, high-bandwidth coil

The planar coil is positioned directly above the diamond sample and attached to a metallic holder, which can be laterally shifted to translate the coil. Due to the thickness of the diamond (500 μm) and the glass slide the minimal vertical stand-off of the coil to the NV centers is ~700 μm. Design parameters of the planar coil, used in our experiments, are listed in Supplementary Table 1. These were found by numerically maximizing the magnetic field at the position of the NV center, located at a planned vertical stand-off of ~700–1000 μm (Supplementary Fig. 1). The coil had an inductance of ≤2.5 μH and a resistance of ≤0.5 Ω. The coil was manufactured by Sibatron AG (Switzerland) and it is mounted onto a copper plate that acts as a heat-sink, using thermally conducting glue. For the coil control, a National Instruments NI PCI 5421 arbitrary waveform generator was used, to generate voltage signals that controlled a waveform amplifier (Accel Instruments TS-250) which drives the coil current.

### Calibration of the coil field Δ**B**

We calibrated the vector field generated by the coil Δ**B** using the target NV, coupled to nuclear spins of interest, and two auxiliary NV centers with different crystallographic orientation. All three NV centers were located in close proximity to each other, with a distance of typically ≤5 μm (Supplementary Fig. 2c). Over this separation the magnetic field of the coil can be assumed to be homogeneous. We determined the orientation of the symmetry axis of many NV centers by moving the permanent magnet over the sample and observing the ODMR splitting. The azimuthal orientation of the target NVs defines the x-axis in the laboratory and NV frame (*ϕ* = 0). This orientation was the same for all target NV centers investigated in this work. The auxiliary NV centers were selected to be oriented along *ϕ*_*a*,1_ = 90° and *ϕ*_*a*,2_ = 270° (Supplementary Fig. 2b). To calibrate the coil field, we removed the permanent magnet and recorded ODMR spectra for the target NV center and both auxiliary NV centers with the field of the coil activated. In this way, we record in total 6 ODMR lines, with 2 lines per NV center.

A numerical, nonlinear optimization method was used to determine the magnetic field Δ**B** from these ODMR resonances. For each of the three NV centers we simultaneously minimized the difference between the measured ODMR lines and the eigenvalues of the ground-state Hamiltonian:

8 $$H_i = DS_z^2 + \gamma _{\mathrm{e}}\left( {{\mathrm{\Delta }}{\bf{B}}} \right)_i \cdot {\bf{S}}.$$

Here, the magnetic field (Δ**B**)_*i*_ acting onto the specific NV center is obtained by a proper rotation of Δ**B** into the respective reference frame.

### Precise alignment of the bias field **B**_0_

Precise alignment of the external bias field to the quantization axis of the NV center (*z*-axis) is critical for azimuthal localization measurements, because residual transverse fields of **B**_0_ modify the precession frequencies in the same way as the field of the coil. The coarse alignment of the magnet and a rough adjustment of the magnitude of the field, to ~10 mT, was achieved by recording ODMR spectra of the target NV center for different (*x*, *y*, *z*)-positions of the magnet. Afterwards, we iteratively optimized the alignment of the magnet. In each iteration, we reconstructed the vector field **B**_0_ acting on the target NV centers using the method used for the calibration of Δ**B**. Subsequently, we moved the magnet in the lateral (*x*, *y*)-plane of the laboratory frame. The direction and step size was determined from a field map of the permanent magnet and the residual transverse components of the field **B**_0_. We terminated this iterative process when the residual transverse field components were smaller than 50 μT.

### Determination of hyperfine couplings (*a*_||_, *a*_⊥_) from (*f*_0_, *f*_−1_, *f*_R_)

The hyperfine couplings *a*_||_ and *a*_⊥_ in the limit $2\pi f_0 \gg a_{||},a_ \bot$ are given by:

9 $$a_{||}{\mathrm{/}}(2\pi ) = f_{ - 1} - f_0.$$

10 $$a_ \bot {\mathrm{/}}(2\pi ) = \pi f_{\mathrm{R}}.$$

In our experiments the hyperfine couplings and the nuclear Larmor frequency *f*_0_ were of similar magnitude, and we used the following transformations^21^ to obtain the hyperfine couplings.

11 $$a_{||} = 2\pi f_{ - 1}\left( {\frac{{{\mathrm{cos}}\left( {2\pi f_{ - 1}\frac{\tau }{2}} \right){\mathrm{cos}}\left( {2\pi f_0\frac{\tau }{2}} \right) - {\mathrm{cos}}\left( {\pi - 2\pi f_{\mathrm{R}}\tau } \right)}}{{{\mathrm{sin}}\left( {2\pi f_{ - 1}\frac{\tau }{2}} \right){\mathrm{sin}}\left( {2\pi f_0\frac{\tau }{2}} \right)}}} \right) - 2\pi f_0.$$

12 $$a_ \bot = \sqrt {\left( {2\pi f_{ - 1}} \right)^2 - \left( {2\pi f_0 + a_{||}} \right)^2}.$$

### Monte Carlo error propagation

Confidence intervals for *ϕ* and *a*_iso_ were obtained using standard Monte Carlo error propagation^36^. The Monte Carlo simulation took calibration uncertainties in the external fields **B**_0_, Δ**B** and in the observed precession frequencies $f_{m_s}$ into account. All parameters subject to uncertainty were assumed to follow a normal distribution. Precession frequencies were determined using a nonlinear, least-squares fitting algorithm and their measurement uncertainties were obtained from the fit error^21^. The uncertainty in the magnetic field components was estimated from the residuals between calculated and measured ODMR lines in the calibration method for **B**_0_, Δ**B**.

### Nuclear g-factor enhancement

The nuclear g-factor enhancement factor *α*(*m*_*S*_) given in Eq. (6) of the main text is based on the approximation of small external bias fields $D \gg \gamma _{\mathrm{e}}B_0$. More generally the *m*_*S*_-dependent enhancement factors are given by^37^:

13 $$\alpha \left( {m_S} \right) = \frac{{\left( {3\left| {m_S} \right| - 2} \right)D + m_S\gamma _{\mathrm{e}}B_0}}{{D^2 - \gamma _{\mathrm{e}}^2B_0^2}}\frac{{\gamma _{\mathrm{e}}}}{{\gamma _{\mathrm{n}}}}\left( {\begin{array}{*{20}{c}} {A_{xx}} & {A_{xy}} & {A_{xz}} \\ {A_{yx}} & {A_{yy}} & {A_{yz}} \\ 0 & 0 & 0 \end{array}} \right),$$

which is also valid in the limit of large magnetic fields $\gamma _{\mathrm{e}}B_0 \gg D$ and provides, in principle, more accurate theory values for small *B*_0_. We have analyzed our experimental data using this expression and found deviations to Eq. (6) that are smaller than the frequency resolution in our experiments.

### Code availability

Custom code was programmed to perform the Monte Carlo simulations. The code is available from the corresponding author upon request.

## Electronic supplementary material

Supplementary Information

## Data Availability

The data that support the findings of this study are available from the corresponding author upon request.
