# Supplementary material for: Three-dimensional localization spectroscopy of individual nuclear spins with sub-Angstrom resolution
Source: Nat Commun. 2018 Nov 8;9:4678. doi: 10.1038/s41467-018-07121-0 (PMC6224602; doi:10.1038/s41467-018-07121-0)
Supplement: Supplementary file 1 — Supplementary Information [file 41467_2018_7121_MOESM1_ESM.pdf]

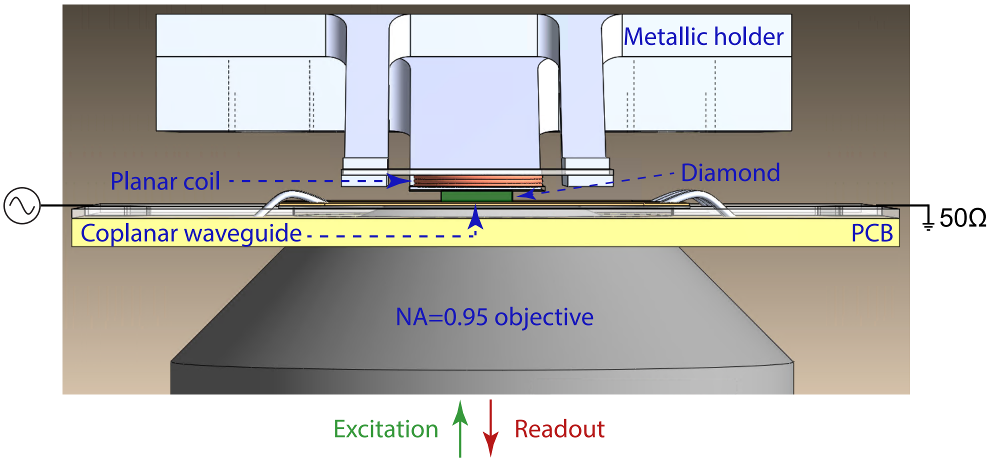

Supplementary Figure 1: Schematic of the experimental setup used in this work. Microwaves for spin control are transmitted through a coplanar waveguide, which has been lithographically defined on a quartz slide. A bulk diamond chip is placed on top of a quartz slide and NV centers are excited and detected with a high-resolution (NA=0.95) objective, which is installed below the quartz slide. The planar coil is located directly on top of the diamond chip and glued to a copper plate, which itself is mounted on metallic holder.

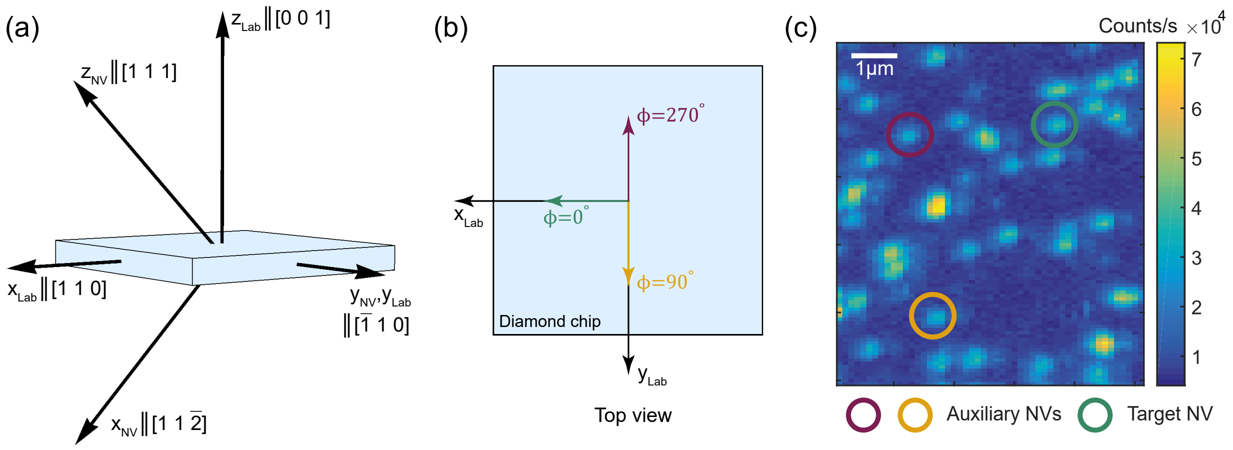

Supplementary Figure 2: (a) Definition of the laboratory and NV center coordinate systems. The diamond chip with $\langle 1\,1\,0 \rangle$ edges and a $\langle 0\,0\,1 \rangle$ front facet is indicated in light blue. The origins of the coordinate systems coincide with the position of the vacancy in the lattice. (b) (Top view) NV centers with laboratory, azimuthal orientation $\phi = 0$ (green) are used for localization spectroscopy. NV centers with azimuthal orientations $\phi_{a,1} = 90°$ (yellow) and $\phi_{a,2} = 270°$ (purple) are employed as auxiliary field sensors for the calibration of vector magnetic fields. (c) Confocal scan of a segment of the diamond chip. A green circle indicates NV2 of the main text and yellow and purple circles highlight the auxiliary NV centers used for field calibrations.

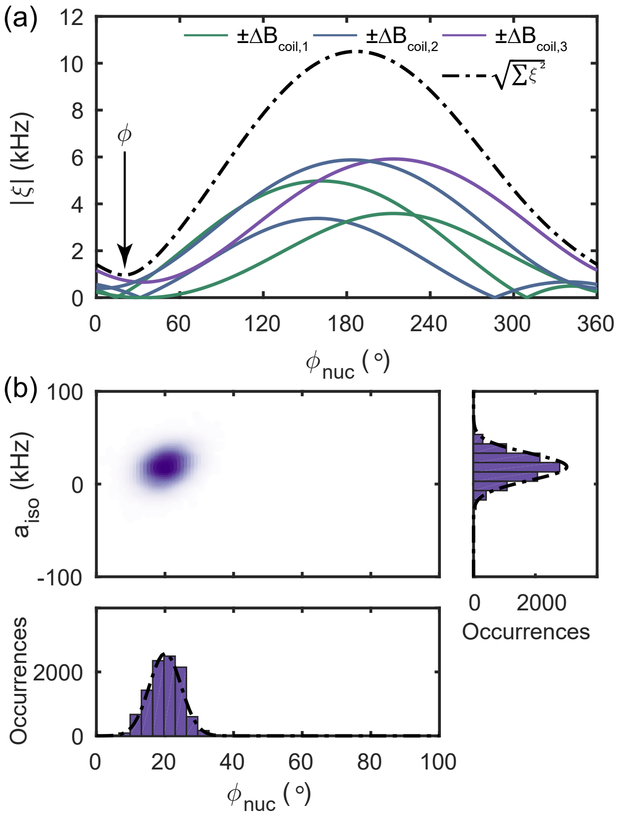

Supplementary Figure 3: Determination of ϕ and $a_{\rm iso}$ for spins $^{13}C_2$. (a) Cost functions $|\xi(\phi)|$ as described in the main manuscript. The maximum likelihood estimate of ϕ is highlighted with an arrow. (b) Scatter density of $(\phi, a_{\rm iso})$ estimates of the Monte Carlo error propagation. Confidence intervals for ϕ and $a_{\rm iso}$ were obtained from the histograms shown at the sides of the scatter density.

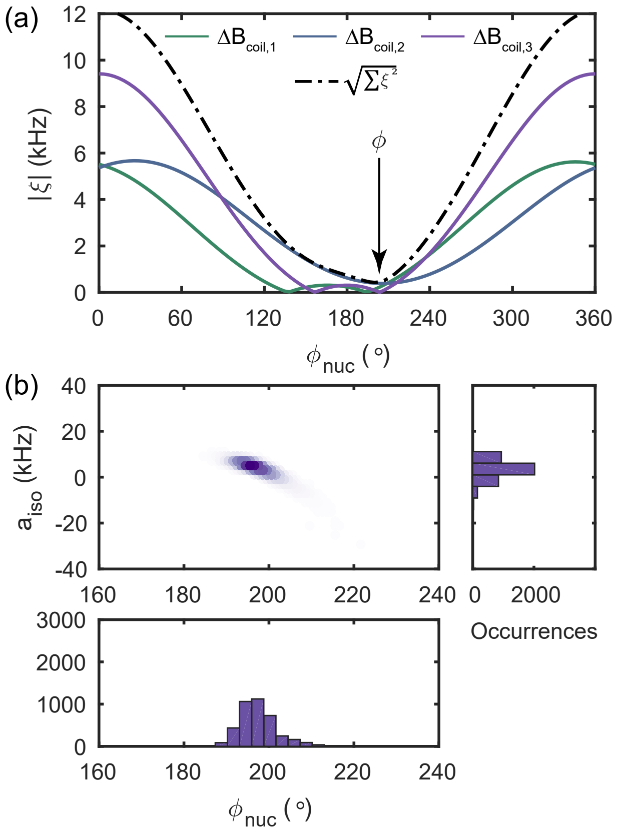

Supplementary Figure 4: Determination of ϕ and a_{iso} for spins $^{13}C_3$. (a) Cost functions $|\xi(\phi)|$ as described in the main manuscript. The maximum likelihood estimate of ϕ is highlighted with an arrow. (b) Scatter density of (ϕ, a_{iso}) estimates of the Monte Carlo error propagation. Confidence intervals for ϕ and a_{iso} were obtained from the histograms shown at the sides of the scatter density.

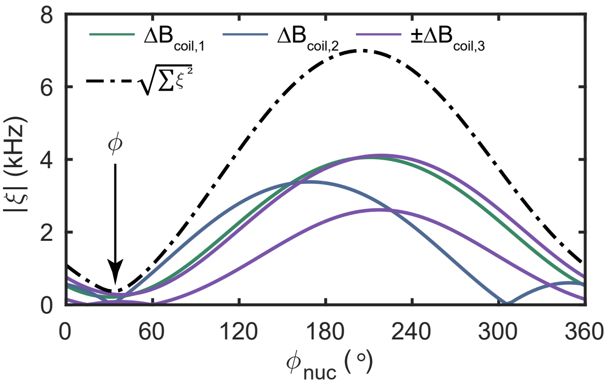

Supplementary Figure 5: Determination of ϕ and a_{iso} for spins $^{13}C_4$. We show the cost functions $|\xi(\phi)|$ as described in the main manuscript. The maximum likelihood estimate of ϕ is highlighted with an arrow.

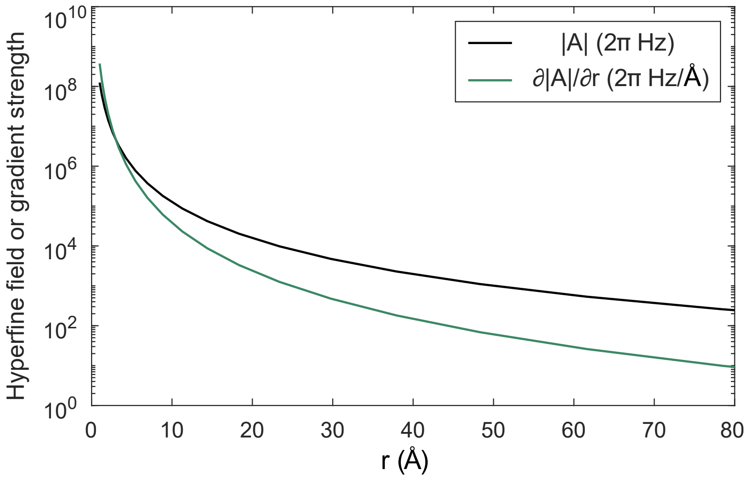

Supplementary Figure 6: Hyperfine field and radial hyperfine gradient of the NV center's electron spin as a function of distance r. The hyperfine field is expressed in angular frequency units $(2\pi \times \mathrm{Hz})$ and the gradient of the hyperfine field is expressed in units of $2\pi \times \mathrm{Hz}/\text{Å}$.

geometry		
	no. of windings (lateral)	9
	no. of layers (vertical)	3
	wire diameter (inc. isolation)	$120\,\mu$m
	inner diameter	2 mm
	outer diameter	4.2 mm
	vertical height	$360\,\mu$m
magnetic fields		
	magnetic field (~ 1 mm vertical offset, simulated)	~ 6.4 mT/A
	magnetic field (~ 1 mm vertical offset, measured)	~ 5.6 mT/A
electrical		
	self-inductance	$\sim 2.5\,\mu$H
	ohmic resistance (at DC)	$\sim 0.5\,\Omega$

Supplementary Table 1: Geometry, magnetic and electrical design properties of the planar coil used in this work.

magnetic field $\vec{B}_0$	$(0.03, -0.06, 9.50)\,\mathrm{mT}$
magnetic field stability	$\leq 10\ \mu\mathrm{T}$
nuclear precession frequency f_0	101.7(1) kHz
nuclear precession frequency f_{-1}	114.2(1) kHz
nuclear rotation frequency f_R	14.4(1) kHz
parallel hyperfine coupling $a_{\|\|}/(2\pi)$	3.1(1) kHz
perpendicular hyperfine coupling $a_{\perp}/(2\pi)$	44.5(1) kHz
CPMG pulse spacing τ	$4.546\,\mu\mathrm{s}$
CPMG pulse number N	4

Supplementary Table 2: Spectroscopy results in aligned magnetic field for $^{13}C_1$

measurement index	f'_0/kHz	f'_{-1}/kHz	$\vec{B}_0$/mT	$\Delta\vec{B}$/mT
#1	88.3(2)	103.2(2)	(0.03,-0.06,9.50)	(-1.72,0.61,-1.55)
#2	117.8(2)	128.1(2)	(0.03,-0.06,9.50)	(1.72,-0.61,1.55)
#3	88.2(2)	107.1(2)	(0.01,0.02,9.41)	(-1.39,-0.92,-1.68)
#4	118.1(2)	125.1(2)	(0.01,0.02,9.41)	(1.39,0.92,1.68)
#5	86.9(2)	104.5(2)	(0.03,-0.04,9.49)	(-1.75,0.07,-1.81)
#6	120.7(1)	129.5(1)	(0.03,-0.04,9.49)	(1.75,-0.07,1.81)

Supplementary Table 3: Spectroscopy results in dynamically tilted magnetic fields for $^{13}C_1$

magnetic field $\vec{B}_0$	$(0.028, -0.056, 9.502)\,\mathrm{mT}$
magnetic field stability	$\leq 10\ \mu$T
nuclear precession frequency f_0	101.1(2) kHz
nuclear precession frequency f_{-1}	230.3(2) kHz
nuclear rotation frequency f_R	12.5(1) kHz
parallel hyperfine coupling $a_{\|\|}/(2\pi)$	119.0(1) kHz
perpendicular hyperfine coupling $a_{\perp}/(2\pi)$	65.9(1) kHz
CPMG pulse spacing τ	$3.032\,\mu$s
CPMG pulse number N	8

Supplementary Table 4: Spectroscopy results in aligned magnetic field for $^{13}C_2$

measurement index	f'_0/kHz	f'_{-1}/kHz	$\vec{B}_0$/mT	$\Delta\vec{B}$/mT
#1	85.2(2)	103.2(2)	(0.03,-0.06,9.50)	(-1.72,0.61,-1.55)
#2	120.4(2)	249.8(3)	(0.03,-0.06,9.50)	(1.72,-0.61,1.55)
#3	83.8(2)	209.8(3)	(0.01,0.02,9.41)	(-1.39,-0.92,-1.68)
#4	121.9(2)	251.8(2)	(0.01,0.02,9.41)	(1.39,0.92,1.68)
#5	82.9(1)	209.0(1)	(0.03,-0.04,9.49)	(-1.75,0.07,-1.81)

Supplementary Table 5: Spectroscopy results in dynamically tilted magnetic fields for $^{13}C_2$

magnetic field $\vec{B}_0$	$(0.05, 0.05, 9.630)\,\mathrm{mT}$
magnetic field stability	$\leq 10\ \mu\mathrm{T}$
nuclear precession frequency f_0	102.8(1) kHz
nuclear precession frequency f_{-1}	127.8(1) kHz
nuclear rotation frequency f_R	13.0(2) kHz
parallel hyperfine coupling $a_{\|\|}/(2\pi)$	18.5(1) kHz
perpendicular hyperfine coupling $a_{\perp}/(2\pi)$	41.4(2) kHz
CPMG pulse spacing τ	$4.596\,\mu\mathrm{s}$
CPMG pulse number N	4

Supplementary Table 6: Spectroscopy results in aligned magnetic field for $^{13}C_3$

measurement index	$f_{-1} - f_0$/kHz	$\vec{B}_0$/mT	$\Delta\vec{B}$/mT
#1	28.0(2)	(0.05,0.01,9.63)	(-1.20,0.24,-1.08)
#2	28.5(2)	(0.05,0.01,9.63)	(-0.99,-0.51,-1.17)
#3	29.9(2)	(0.06,0.01,9.63)	(-1.83,-0.06,-1.75)

Supplementary Table 7: Spectroscopy results in dynamically tilted magnetic fields for $^{13}C_3$

magnetic field $\vec{B}_0$	$(-0.0592, -0.001, 9.615)\,\mathrm{mT}$
magnetic field stability	$\leq 10\ \mu$T
nuclear precession frequency f_0	102.9(1) kHz
nuclear precession frequency f_{-1}	106.5(1) kHz
nuclear rotation frequency f_R	6.3(2) kHz
parallel hyperfine coupling $a_{\|\|}/(2\pi)$	1.9(1) kHz
perpendicular hyperfine coupling $a_{\perp}/(2\pi)$	19.2(1) kHz
CPMG pulse spacing τ	$4.876\,\mu$s
CPMG pulse number N	8

Supplementary Table 8: Spectroscopy results in aligned magnetic field for $^{13}C_4$

measurement index	$f_{-1} - f_0$/kHz	$\vec{B}_0$/mT	$\Delta\vec{B}$/mT
#1	1.6(1)	(-0.06,-0.00,9.62)	(-0.10,-0.63,-1.08)
#2	2.3(1)	(-0.06,-0.00,9.62)	(-1.20,0.247,-1.08)
#3	1.5(3)	(-0.04,-0.05,9.77)	(-0.97,-0.75,-0.94)
#4	4.6(3)	(0.06,0.01,9.64)	(0.97,0.75,0.94)

Supplementary Table 9: Spectroscopy results in dynamically tilted magnetic fields for $^{13}C_4$
